# CD74 is associated with inflamed tumor immune microenvironment and predicts responsiveness to PD-1/CTLA-4 bispecific antibody in patients with solid tumors

**DOI:** 10.1007/s00262-023-03604-2

**Published:** 2024-01-27

**Authors:** Jianghua Wang, Xiaoting Li, Guanxi Xiao, Jayesh Desai, Sophia Frentzas, Zhongmin Maxwell Wang, Yu Xia, Baiyong Li

**Affiliations:** 1 Research and Development Department, Akeso Biopharma Inc, Zhongshan, Guangdong China; 2 Department of Oncology, Sir Peter MacCallum, The University of Melbourne, Parkville, VIC Australia; 3 Department of Medical Oncology, Monash Health, Melbourne, VIC Australia; 4 Faculty of Medicine, Nursing and Health Sciences, Monash University, Melbourne, VIC Australia; 5 Procurement and Sourcing Department and Clinical Operation Department, Akeso Biopharma Inc, Zhongshan, Guangdong China; 6 President Office, Akeso Biopharma Inc, Zhongshan, Guangdong China

**Keywords:** CD74, PD-1/CTLA-4 bispecific antibody, Biomarker, Tumor microenvironment, Immune subtypes

## Abstract

**Introduction:**

Cadonilimab (AK104) is a first-in-class tetravalent bispecific antibody that targets both PD-1 and CTLA-4, showing a manageable safety profile and favorable clinical benefits. This study aimed to identify the biomarkers of clinical response and explore the immune response within the tumor microenvironment upon the AK104 therapy in advanced solid tumors.

**Material and methods:**

Gene expression profiles of paired pre- and post-treatment tumor tissues from twenty-one patients were analyzed. The association of gene expression levels with either clinical efficacy or prognosis was evaluated and subsequently validated with published datasets using log-rank for Kaplan–Meier estimates. Comparative immune profile analyses of tumor microenvironment before and after AK104 treatment were conducted. The visualization of tumor-infiltrating lymphocytes was performed using multiplex immunohistochemistry. The predictive value of CD74 was further validated with protein expression by immunohistochemistry.

**Results:**

Baseline *CD74* gene expression was associated with favorable patient outcomes (overall survival [OS], HR = 0.33, 95% CI 0.11–1.03, *p* = 0.0463), which was further confirmed with the published datasets. Tumors with high *CD74* gene expression at baseline were more likely to exhibit an immune-inflamed microenvironment. AK104 efficiently enhanced the infiltration of immune cells in the tumor microenvironment. Additionally, high CD74 protein expression (≥ 10% of the tumor area occupied by CD74 stained immune cells) at baseline was associated with better progressive-free survival (HR = 0.21, 95% CI 0.06–0.68, *p* = 0.0065) and OS (HR = 0.35, 95% CI 0.12–1.08, *p* = 0.0615).

**Conclusions:**

Our findings demonstrate that CD74 is a promising predictive biomarker for AK104 therapeutic response in advanced solid tumors.

*Trial registration number* NCT03261011.

**Supplementary Information:**

The online version contains supplementary material available at 10.1007/s00262-023-03604-2.

## Introduction

Immuno-oncology therapies have achieved great success in clinics with the widespread application of immune checkpoint antibodies in numerous types of tumors. The programmed death receptor 1 (PD-1), the ligand of PD-1 (PD-L1) and cytotoxic T-cell lymphocyte-associated protein 4 (CTLA-4) are the major immune checkpoint molecules involved in immune surveillance escape during tumorigenesis [1, 2]. The bindings of PD-1 and CTLA-4, expressed on activated T cells, to their ligands on tumor cells (PD-L1) or antigen-presenting cells (APCs) (CD80/CD86) restrain the anti-tumor T cell activities. The immunotherapeutic antibodies directed against these immune checkpoint molecules, such as nivolumab, ipilimumab, and pembrolizumab, reactivate the cytotoxic T cells to eradicate tumor cells via blocking the PD-L1/PD-1 or CTLA-4-CD80/CD86 signaling pathways [3].

Treatment with a combination of immune checkpoint inhibitors (ICIs) (e.g., anti-PD-1/L1 and anti-CTLA-4) offers the potential for superior efficacy compared with either therapy alone [4, 5]. However, traditional predictive biomarkers in ICI monotherapy, such as PD-L1 and tumor mutation burden (TMB), do not appear to be reliable in predicting the treatment response to ICI combination therapy [6, 7]. For example, in Checkmate 227 study, nivolumab (anti-PD-1) plus ipilimumab (anti-CTLA-4) achieved durable clinical benefit in all patients regardless of tumor PD-L1 expression [8]. In CA209-538, TMB did not show a meaningful predictive value in cancer patients treated with nivolumab and ipilimumab, whereas high blood TMB was associated with favorable clinical benefit with durvalumab (anti-PD-L1) and tremelimumab (anti-CTLA-4) versus chemotherapy in MYSTIC clinical trial [9, 10]. Therefore, there is a need to identify a robust and precise biomarker that can predict which patients are most likely to benefit from the combination of ICIs treatment.

Cadonilimab (AK104) is a novel tetravalent PD-1/CTLA-4 bispecific antibody. It demonstrates a tolerable safety profile and favorable clinical response in various types of tumors, such as cervical cancer and gastric cancer [11–13]. The molecular mechanisms of regulation in the tumor microenvironment in patients treated with AK104 are not fully understood. In this study, we report the results of a prospective multicenter study of the COMPASSION-01 trial (NCT03261011) using NanoString nCounter and multiplex immunohistochemistry (mIHC). This work is the first to explore the immune modulation mechanisms and the potential predictive biomarker in patients with solid tumors upon AK104 treatment.

## Materials and methods

### Study design and sample collection

The COMPASSION-01 trial (NCT03261011) was a phase Ia/Ib multicenter, open-label study to evaluate the safety, pharmacokinetics, and anti-tumor activity of AK104 in subjects with advanced solid tumors [14]. The subject population included male or female adults, aged ≥ 18 years, with adequate organ function, an Eastern Cooperative Oncology Group (ECOG) performance score of 0–1, and histologically or cytologically documented advanced or metastatic solid tumor that is relapsed or refractory to standard therapies, or for which no effective standard therapy is available. This study is comprised of 2 phases: dose-escalation (phase Ia) and dose-expansion (phase Ib). Patients from six study centers in Australia received AK104 at doses of 0.2, 0.5, 1.0, 2.0, 4.0, 6.0, 10.0 mg/kg Q2W, 450 mg Q2W, and 15.0, 25.0 mg/kg Q3W. Tumor responses were evaluated according to Response Evaluation Criteria in Solid Tumors Version 1.1 (RECIST 1.1) criteria according to the protocol schedule of activities (every 8 weeks). Based on the best overall response (BOR), clinical efficacy was classified as disease control (DC) (i.e., complete response [CR], partial response [PR], or stable disease [SD]) or progressive disease (PD).

Thirty-eight formalin-fixed paraffin-embedded (FFPE) tumor tissues of 21 patients from five clinical centers were collected pre- or post-treatment via tumor biopsy. The collection of tumor samples and access to clinical data for research were approved by Bellberry and Royal Melbourne Hospital (initially Melbourne Health) Human Research Ethics Committee. This clinical trial was conducted in accordance with the Declaration of Helsinki, International Conference on Harmonisation Guidelines for Good Clinical Practice, and applicable laws and regulations. All patients provided written informed consent forms.

### NanoString nCounter gene expression assay

A total of 38 specimens were of sufficiently high quality for RNA evaluation. For each sample, 70 ng or more than 70 ng (calculate using formula 1, where target input is 70 ng and % [50–300 nt] is the percentage of fragment [50–300 nt]) of total RNA was used to measure the expression of 730 immune-related genes and 40 housekeeping genes using the nCounter platform (NanoString Technologies; Seattle, Washington, USA) [15] and the PanCancer Immune Profiling Panel according to the manufacturer’s instructions. After data quality control, 193 genes with low signal (below the limit of detection) were removed, and 537 immune-related genes were used for subsequent analysis. Data were normalized to the housekeeping genes using nSolver software (version 4.0) and transformed to log2.

1 $$\frac{{{\text{Target}}\;{\text{Input}}}}{{1 - \% \;\left( {50 - 300\;{\text{nt}}} \right)}}={\text{Adjusted}}\;{\text{Input}}$$

### mIHC

mIHC staining of FFPE tumor sections was performed using PANO Multiplex IHC kit (Cat# 0003100100, Panovue) according to the manufacturer’s instructions. To explore the cellular composition and functional states in the tumor microenvironment, we designed a panel of four antibodies for T cell subtyping: Foxp3 antibody (Cat# BLG320202, BioLegend), CD4 antibody (Cat# ZM0418, Zsbio), CD8A antibody (Cat# CST70306, Cell Signaling Technology), and Ki67 antibody (Cat# BX50040, Biolynx). Briefly, sections were deparaffinized and rehydrated, followed by antigen retrieval using AR9 buffer (pH = 9). After protein blocking, slides were incubated with primary antibody, followed by horseradish peroxidase (HRP)-conjugated secondary antibody incubation and tyramide signal amplification (TSA). Afterward, the slides were treated with microwave heat to remove the antibody complexes. The same sequence of blocking, primary antibody incubation, HRP antibody incubation, and TSA was repeated for each marker. After four sequential reactions, slides were counterstained with DAPI and scanned to acquire multispectral images using the Mantra System (PerkinElmer), which captures the fluorescent spectra at 20-nm wavelength intervals from 420 to 720 nm with identical exposure time. For each slide, more than three fields were selected for image capture. The quantitative analysis of positive rates for specific cell types (CD4^+^–CD4 T cells, CD8^+^–CD8 T cells, CD4^+^Foxp3^+^-regulatory T cells [Tregs], CD4^+^Ki67^+^-proliferating CD4 T cells, and CD8^+^Ki67^+^-proliferating CD8 T cells) [16–18] was performed using the InForm software (V2.4).

### Immunohistochemistry (IHC)

CD74 protein expression in collected tumor tissues at baseline was analyzed using an IHC standard process. Slides were deparaffinized, rehydrated, and antigen retrieved, followed by staining with CD74 antibody (Cat# CST77274, Cell Signaling Technology). The bound HRP antibody was detected using the DAB solution (Cat# DA1010, Solarbio Life Science). Slides were counterstained with hematoxylin and bluing reagent. High-resolution digital images were obtained using Olympus OlyVIA. CD74 protein expression was determined using ICP, which was the percentage of tumor area occupied by CD74 stained immune cells. The interpretation of ICP was conducted by one pathologist.

### Differential expression gene (DEG) analysis

The DEG analysis was conducted to compare patients with response differences (DC or PD) using the NanoStringDiff package [19], which uses a generalized linear model of the negative binomial family to characterize count data and allows for multifactor design. The DEGs with a *p*-value < 0.05, and absolute fold-change ≥ 2 were included in further analysis for predictor exploration.

### Functional enrichment analysis

Gene Set Enrichment Analysis (GSEA) [20], an algorithm widely used to identify statistically enriched pathways in ranked gene lists, was performed using the R package clusterProfiler [21]. The normalized enrichment score (NES) was used to evaluate the degree of enrichment of the gene set. In this study, NES showing a positive number means that the gene set is enriched in the response (DC) group, whereas a negative number indicates that the gene set is enriched in the progression (PD) group.

### Immune pathway and immune cell analysis

The PanCancer Immune Profiling Panel contains many features of the immune response, including 22 immune-related pathways and 14 immune cell types. In addition, other immune-related signatures, such as interferon-γ (IFN-γ) gene set (*IFI16*, *IFI27*, *MX1*, *IFNG*, *STAT1*, *CCR5*, *CXCL9*, *CXCL10*, *CXCL11*, *IDO1*, *PRF1*, *GZMA*, and *HLA-DRA*), human leukocyte antigen-I (HLA-I) gene set (*HLA-A*, *HLA-B*, and *HLA-C*), and HLA-II gene set (*HLA-DMA*, *HLA-DMB*, *HLA-DPA1*, *HLA-DPB1*, and *HLA-DRA*), were used to facilitate a more comprehensive analysis of the tumor immune microenvironment. These immune scores were used to characterize the average expression level of the signatures by calculating the geometric mean.

### Immune subtype identification

The ConsensusClusterPlus R package [22] was used to perform clustering analysis to identify immune-related molecule subtypes based on 22 immune-related signatures, including B cells, T cells, Th1 cells, CD8 T cells, Tregs, exhausted CD8 T cells, cytotoxic cells, dendritic cells, macrophages, mast cells, neutrophils, natural killer (NK) cells, CD56dim NK cells, IFN-γ, HLA-I, HLA-II, adhesion, antigen processing, cancer/testis (CT) antigen, B cell functions, leukocyte functions, and senescence. The parameters were 200 iterations, 80% resampling rate, Pearson correlation, and k-means clustering algorithm.

### Validation analysis

We collated validation datasets from the supplementary data of previously published immunotherapy clinical cohorts that contained RNA data and clinical information, including overall survival (OS), progressive-free survival (PFS), and BOR. Clinical efficacy was classified as DC (CR, PR, or SD) or PD. Forty-seven melanoma patients in Liu's cohort [23] and forty-eight patients with melanoma, non-squamous non-small cell lung cancer (NSCLC), squamous NSCLC, or head and neck squamous cell cancer (HNSCC) in Prat's cohort [24] were used for the validation analysis. Patients were divided into high and low *CD74*, *CREB5*, *CD200*, or *STGAL1* expression groups based on the median gene expression levels.

In addition, the transcriptome data and clinical data for 33 cancer types from The Cancer Genome Atlas (TCGA) were downloaded from the UCSC Xena browser (https://xenabrowser.net/) [25]. The association between survival and *CD74* expression of 33 cancer types was performed, and only those cancer types showing significant differences were shown. Correlation analysis between *CD74* and *CD274* (PD-L1 coding gene) or *CTLA4* expression was conducted for all 33 cancer types.

### Statistical analysis

All statistical analyses were performed using R software (version 4.1.0). The significance of differences between groups was estimated by Fisher’s exact test, Wilcoxon test, or Wald test, as appropriate. All statistical tests were two-sided. PFS was defined as the time from randomization to the first evidence of PD or death from any cause, while OS was defined as the time from randomization to death from any cause. Disease control rate (DCR) was defined as the percentage of patients who achieved CR, PR, or SD. Receiver operating characteristic (ROC) curves were used to evaluate the predictive value of candidate genes or CD74 protein expression quantitative indicators. The area under ROC curves (AUC) can be compared as an indicator of the merits and disadvantages of the model. Hazard ratios (HR) and 95% confidence intervals (CI) were estimated using Univariate Cox proportional hazards regression models. Survival analyses were performed by Kaplan–Meier curves with the log-rank test. *p*-values < 0.05 were considered to indicate statistical significance (*p < 0.05; **p < 0.01; ***p < 0.001; ****p < 0.0001).

## Results

### Clinicopathological characteristics of the study cohort

In this study, 21 patients were selected and examined for exploratory biomarker analyses from COMPASSION-01 trial (NCT03261011), which was designed as a single-arm study and aimed at evaluating the anti-tumor activity of AK104 in patients with advanced solid tumors. Pre-treatment tumor tissues were collected from the 21 patients who received AK104 with the dosing over 6 mg/kg, while 17 of them provided post-treatment (at day 29 post-treatment) tumor tissues (Supplementary Fig. 1). A total of twelve types of tumors were collected. The clinicopathological characteristics of these 21 patients are summarized in Table 1.

**Table 1:** Patient demographics and characteristics at baseline (Until Aug 27, 2021)

Characteristics	Overall (*n* = 21)
Age, median (range)	67 (48, 85)
BMI, median (range)	27.42 (18.14, 39.89)
*Gender (%)*	
Female	10 (47.6)
Male	11 (52.4)
*Race (%)*	
White	20 (95.2)
Asian	1 (4.8)
*Dose levels (%)*	
6 mg/kg Q2W	13 (61.9)
450 mg Q2W	7 (33.3)
15 mg/kg Q3W	1 (4.8)
*Clinical stage (%)*	
Stage III	5 (23.8)
Stage IV	15 (71.4)
Unknown	1 (4.8)
*ECOG (%)*	
0	11 (52.4)
1	10 (47.6)
*Smoking status (%)*	
Current	2 (9.5)
Former	6 (28.6)
Never	13 (61.9)
*BOR (%)*	
PR	2 (9.5)
SD	10 (47.6)
PD	9 (42.9)

*BMI* body mass index, *BOR* best overall response, *ECOG* Eastern Cooperative Oncology Group, *PR* partial response, *PD* progressive disease, *SD* stable disease, *Q2W* once every 2 weeks, *Q3W* once every 3 weeks

### Baseline *CD74* mRNA levels and clinical outcomes

**Fig. 1:**
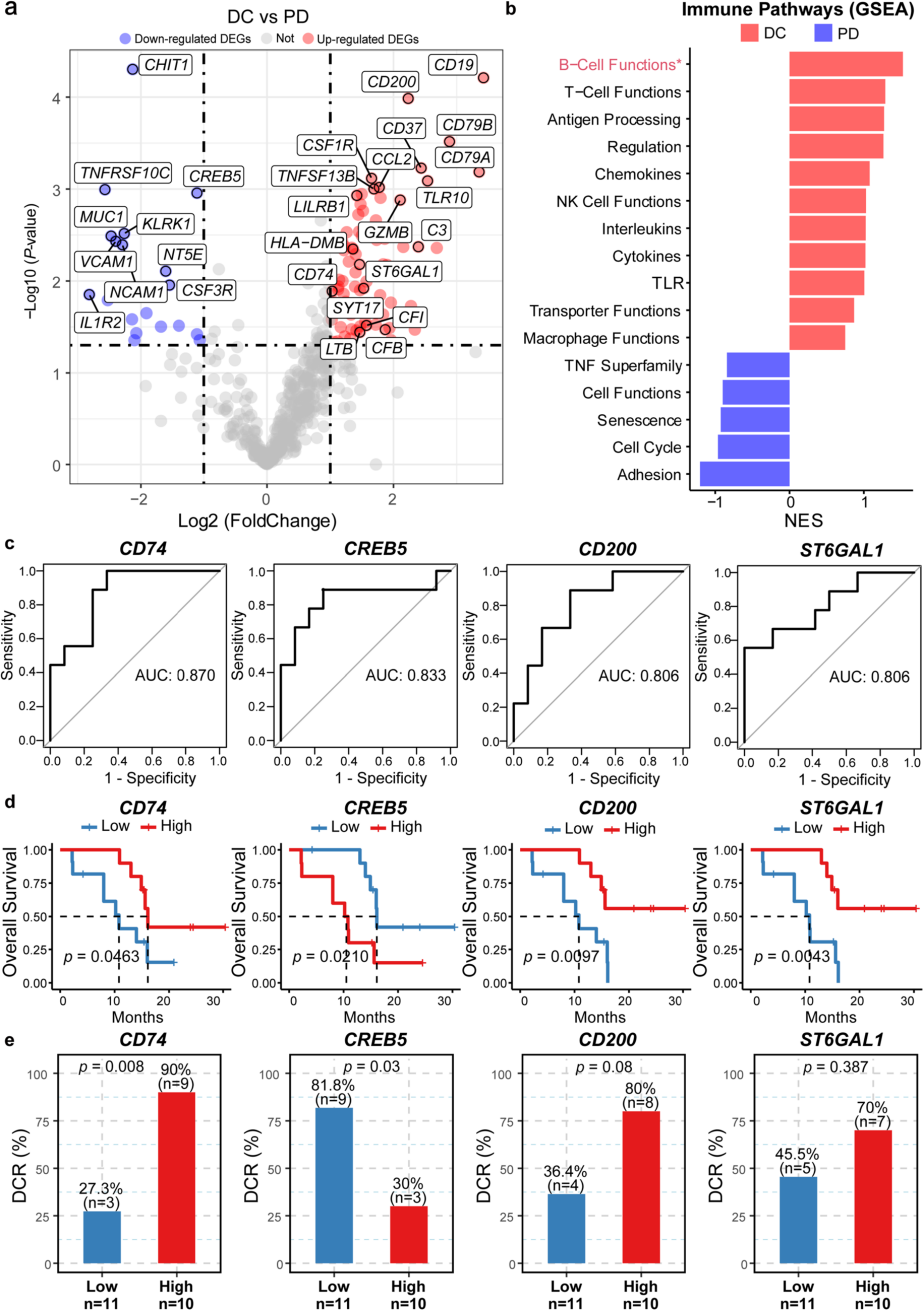
Baseline *CD74* mRNA levels predict clinical response**.** DEG (**a**) and immune pathway (**b**) analysis of baseline tumor tissue samples between DC (*n* = 12) and PD (*n* = 9) groups. Red and Blue dots represent the significantly up-regulated and down-regulated genes, respectively. **c** The predictive effects of *CD74*, *CREB5*, *CD200*, *or STGAL1* to clinical response were analyzed by ROC curve. **d** Kaplan–Meier survival curve for estimating OS based on *CD74, CREB5*, *CD200*, *or STGAL1* mRNA expression (high gene expression group, *n* = 10; low gene expression group, *n* = 11). **e** Bar plot of DCR of *CD74, CREB5*, *CD200*, *or STGAL1* mRNA expression. *p*-values were calculated via Kaplan–Meier curves with the log-rank test (**d**) and Fisher’s exact test (**e**). DC, disease control; GSEA, Gene Set Enrichment Analysis; NES, normalized enrichment score; NK, natural killer; PD, progressive disease; TLR, toll-like receptor; TNF, tumor necrosis factor; DCR, disease control rate

We firstly analyzed the baseline gene expression to identify the potential predictive biomarker. A significantly differential gene expression pattern was observed. Compared to the PD group (*n* = 9), 89 genes were significantly up-regulated and 19 genes were significantly down-regulated in the DC group (PR or SD, *n* = 12) (Fig. 1a). The up-regulated genes were related to B cell function (e.g., *CD19* and *CD79B*), T cell function (e.g., *GZMB* and *LILRB1*), and antigen presentation (e.g., *HLA-DMB* and *CD74*). The result of GSEA further revealed that the most highly enriched gene sets in the DC group were involved in B/T/NK cell functions, antigen processing, and regulation pathways, whereas the PD group mainly exhibited enrichment of adhesion, cell cycle, and senescence gene sets (Fig. 1b).

We next investigated the associations of these differentially expressed genes with clinical response using the ROC curve. Four genes were found as potential candidates to predict the anti-tumoral activity of AK104, including *CD74* (AUC = 0.870), *CREB5* (AUC = 0.833), *CD200* (AUC = 0.806), and *ST6GAL1* (AUC = 0.806) (Fig. 1c). According to the median gene expression levels, patients were divided into high and low gene expression groups for each gene. Patients with high expression levels of *CD74*, *CD200*, or *ST6GAL1* had significantly prolonged OS (*CD74*, HR = 0.33, 95% CI 0.11–1.03, *p* = 0.0463; *CD200*, HR = 0.23, 95% CI 0.07–0.77, *p* = 0.0097; *ST6GAL1*, HR = 0.20, 95% CI 0.06–0.67, *p* = 0.0043; Fig. 1d). However, an opposite trend was observed between *CREB5* gene expression and OS (HR = 3.59, 95% CI 1.14–11.28, *p* = 0.0210). In addition, the elevated intratumoral *CD74* (*p* = 0.008) and decreased *CREB5* (*p* = 0.03) expressions were significantly associated with higher DCR, but no correlation with DCR was observed for *CD200* (*p* = 0.08) or *ST6GAL1* (*p* = 0.387) (Fig. 1e).

To validate the predictive performance of these four genes, we further analyzed the RNA data from another two clinical trials (Liu’s cohort and Prat’s cohort) treated with anti-PD-1 antibodies (pembrolizumab or nivolumab). In Liu's cohort, *CD74* or *ST6GAL1* gene expression was significantly correlated with better PFS (*CD74*, HR = 0.36, 95% CI 0.18–0.74, *p* = 0.0040; *ST6GAL1*, HR = 1.96, 95% CI 1.01–3.79, *p* = 0.0420) and OS (*CD74*, HR = 0.49, 95% CI 0.24–1.01, *p* = 0.0494; *ST6GAL1*, HR = 2.40, 95% CI 1.15–5.03, *p* = 0.0166) (Supplementary Table 1**)**. In Prat’s cohort, the higher *CD74* gene expression was more likely associated with prolonged PFS (HR = 0.51, 95% CI 0.25–1.03, *p* = 0.0578) (Supplementary Table 2**)**. Furthermore, stratification of patients from TCGA database based on *CD74* gene expression indicated a significant association between expression and OS in BLCA, BRCA, LUAD, SKCM, and UCEC (Supplementary Fig. 2).

Taken together, our results suggest that the gene expression level of *CD74* serves as a potential predictor in multiple cancer types for anti-PD-1/CTLA-4 immunotherapy.

### Association between *CD74* and tumor immune microenvironment

**Fig. 2:**
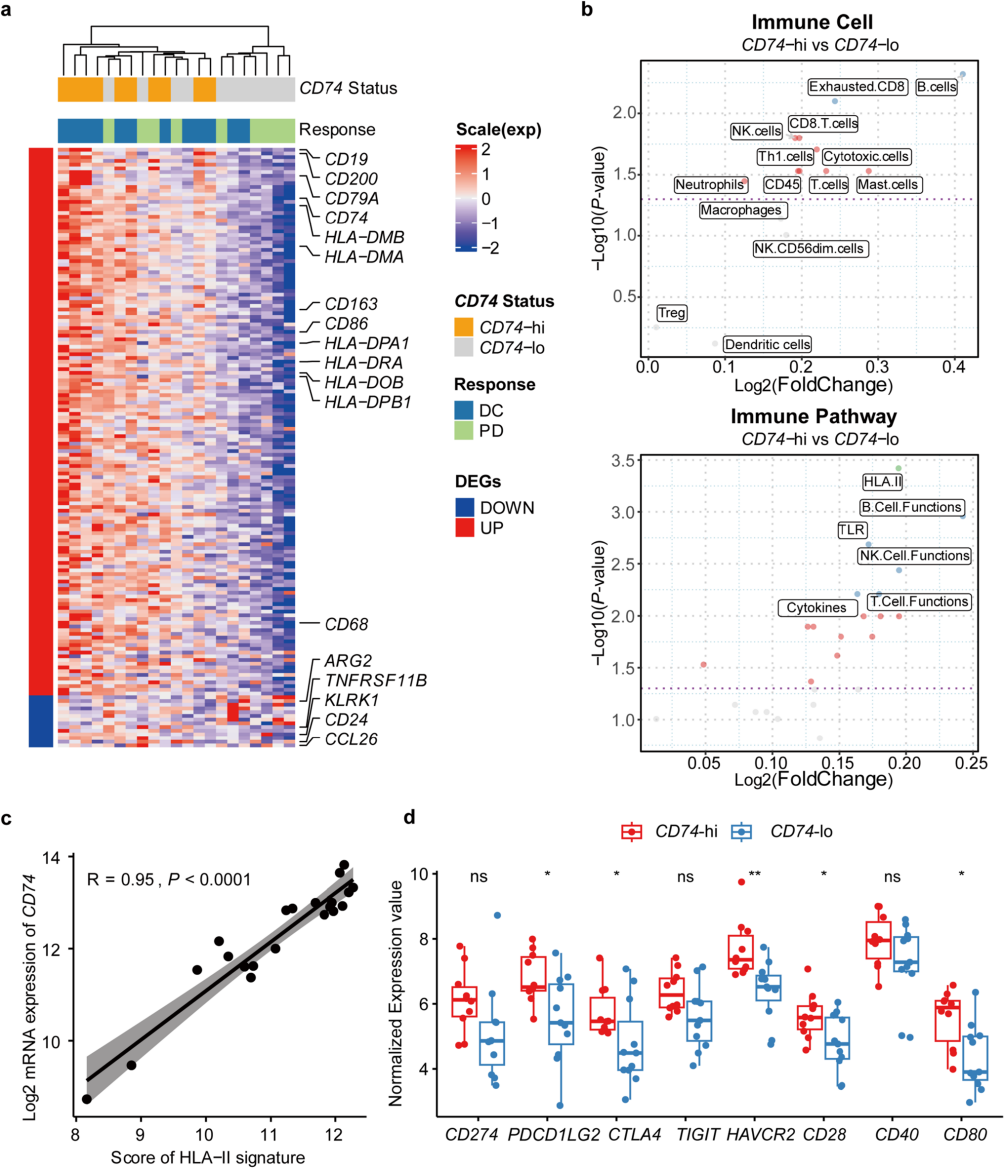
High *CD74* expression is associated with strong anti-tumor immunity in tumor microenvironment. **a** DEG expression levels between the *CD74*-hi (*n* = 10) and the *CD74*-lo (*n* = 11) groups. **b** Immune cells (top) and immune pathway (bottom) analysis of baseline tumor tissue samples between *CD74*-hi (*n* = 10) and *CD74*-lo (*n* = 11) groups. Green, Blue, and Red dots represent significant differences. Green: *p* < 0.001; Blue: *p* < 0.01; Red: *p* < 0.05. **c** Correlation analysis between *CD74* expression levels and HLA-II gene signature scores. **d** Expression levels of immune checkpoint molecules and costimulatory molecules in *CD74*-hi (*n* = 10) and *CD74*-lo (*n* = 11) groups. *p*-values were calculated via Wilcoxon test (**b** and **d**) or Pearson correlation coefficient test (**c**). * p < 0.05, ** *p* < 0.01, ns indicates not significant. *CD74*-hi, *CD74*-high; *CD74*-lo, *CD74*-low; DC, disease control; HLA, human leukocyte antigen; NK, natural killer; PD, progressive disease; Th1 cells, type 1 T helper cells; TLR, toll-like receptor

Having confirmed that *CD74* gene expression was associated with favorable OS, we next investigated the underlying correlation between *CD74* gene expression status and the tumor microenvironment. We compared the baseline GEP of patients in the *CD74* high expression group (*CD74*-hi) and *CD74* low expression group (*CD74*-lo). 147 genes and 14 genes were found significantly up-regulated in *CD74*-hi and *CD74*-lo groups, respectively (Fig. 2a). In terms of immune cells, patients in *CD74*-hi group were illustrated a pattern with a greater infiltration of B cells, exhausted CD8 T cells, NK cells, CD8 T cells, and cytotoxic cells compared to *CD74*-lo group (Fig. 2b, top). The most highly enriched immune pathways in *CD74*-hi group were also primarily associated with immune response, including HLA-II, B cell function, Toll-like receptor (TLR), T cell/NK cell functions, and cytokines (Fig. 2b, bottom). Notably, the expression of *CD74* was positively correlated with HLA-II (Pearson correlation: *R* = 0.95, *p* < 0.0001) (Fig. 2c), which was consistent with previous report that *CD74* was involved in antigen presentation [26–28].

The correlations between gene expression of *CD74* and the binding targets of AK104 (*CD274* and *CTLA4*) were further explored. Intriguingly, compared to *CD74*-lo tumors, *CD74*-hi tumors exhibited significantly higher expression levels of immune checkpoint molecules, such as *CD274* (*p* = 0.051), *PDCD1LG2* (PD-L2 coding gene, *p* = 0.043), *CTLA4* (*p* = 0.036), *HAVCR2* (TIM3 coding gene, *p* = 0.002), *CD28* (known as a counteractor of CTLA-4, *p* = 0.043) and its ligand *CD80* (*p* = 0.016) (Fig. 2d), which may contribute to AK104 responses. The expression of *CD74* was highly correlated with *CD274* (Pearson correlation: *R* = 0.43, *p* = 0.05) and *CTLA4* (Pearson correlation: *R* = 0.68, *p* = 0.00067) (Supplementary Fig. 3a). Consistent with our findings, similar results were observed for multiple cancer types when analyzing the RNA data from TCGA database (Supplementary Fig. 3b).

Collectively, these findings demonstrate that tumors with high *CD74* expression exhibit immune-inflamed microenvironment and higher levels of immune checkpoint-related molecules, showing potential to be more sensitive to immune checkpoint immunotherapy (e.g., PD-1 and CTLA-4).

### The modulation of tumor microenvironment upon AK104 treatment

**Fig. 3:**
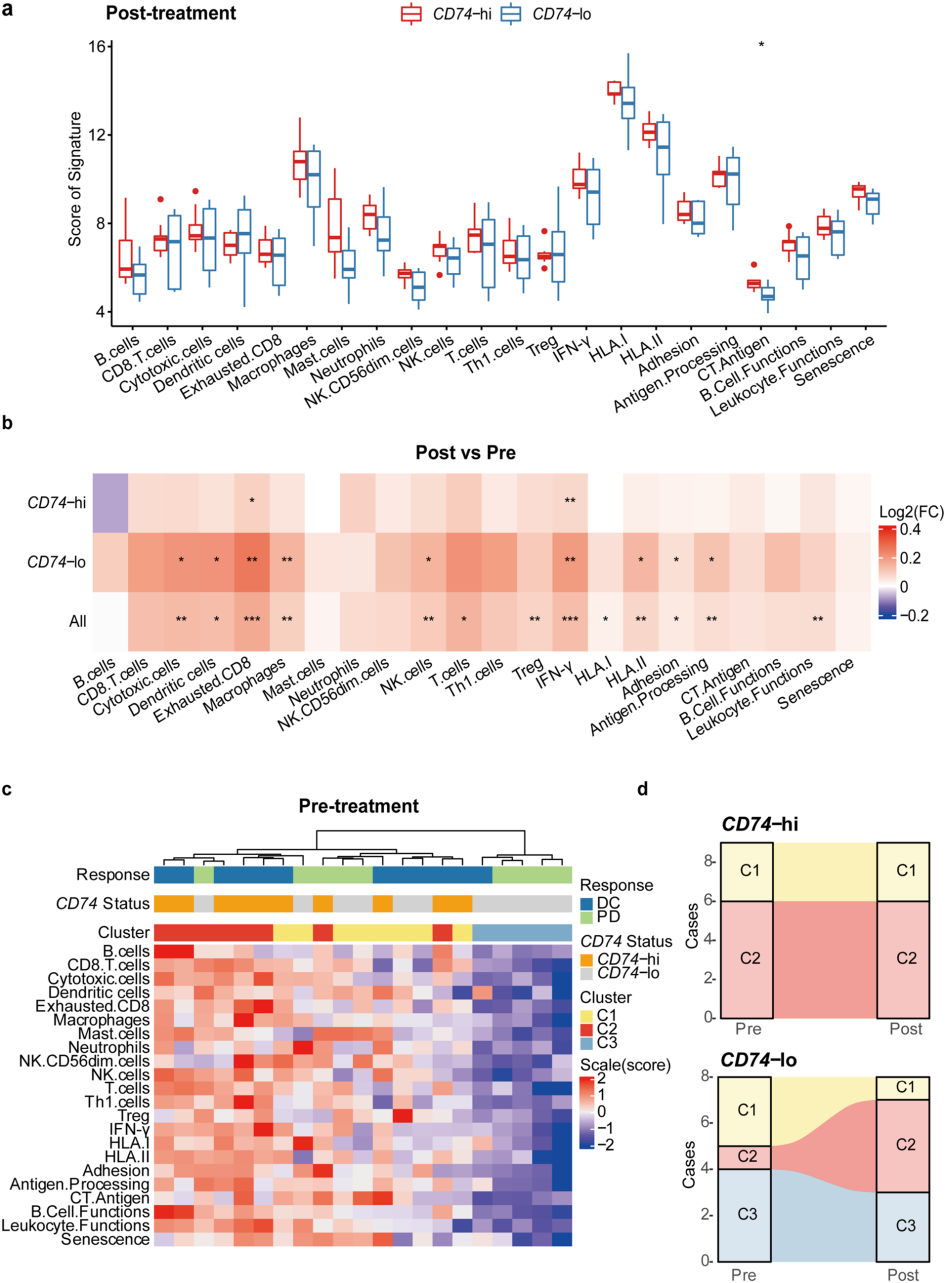
Modulation of immune contexture in *CD74-*hi/-lo groups. **a** Expression levels of immune cells and immune pathways in *CD74*-hi (*n* = 9) or *CD74*-lo (*n* = 8) groups after AK104 treatment. **b** Heatmap for changes of immune cells and immune pathway scores in *CD74*-hi (*n* = 9) or *CD74*-lo (*n* = 8) groups. **c** Heatmap shows 22 immune-related signatures landscapes of three immune subtypes in baseline tumor samples. C1, C2 and C3 indicate intermediate, enriched, and poor immune status, respectively. **d** Changes of immune subtypes in *CD74*-hi (*n* = 9) or *CD74*-lo (*n* = 8) groups following AK104 treatment. *p*-values were calculated via Wilcoxon test. *p < 0.05, **p < 0.01, ***p < 0.001, ns indicates not significant. C1, cluster 1; C2, cluster 2; C3, cluster 3; *CD74*-hi, *CD74*-high; *CD74*-lo, *CD74*-low; CT, cancer/testis; DC, disease control; FC, fold change; HLA, human leukocyte antigen; IFN-γ, interferon-γ; NK, natural killer; PD, progressive disease; Post, post-treatment; Pre, pre-treatment; Th1 cells, type 1 T helper cells; TLR, toll-like receptor; Treg, regulatory T cell

To better understand how AK104 regulates the tumor microenvironment, a total of 17 paired baseline and AK104 post-treatment tumor samples were collected and analyzed for GEP. Higher gene signature scores of immune cells and immune pathways were observed in *CD74*-hi group (*n* = 9) than in *CD74*-lo group (*n* = 8) in post-treatment tumors (Fig. 3a), which were similar to the results described previously at baseline (Fig. 2b). When monitoring the dynamic changes between pre- and post-treatment, we found AK104 activated the tumor microenvironment with significantly increased gene expressions of immune cells (e.g., exhausted CD8 T cells, cytotoxic cells, and macrophages) and immune pathways (e.g., IFN-γ, HLA-I/II, and antigen processing) for all patients (Fig. 3b). Intriguingly, significant increases of different immune cells and enriched pathway gene sets were observed in *CD74*-lo group, whereas *CD74*-hi group exhibited only a significant increase of exhausted CD8 T cells and enriched IFN-γ pathway gene set. The above results suggest that AK104 could efficiently promote the activation of tumor immune microenvironment regardless of *CD74* gene expression. Despite the considerable increase of immune parameters in *CD74*-lo group, the poor response might be attributed to the relatively lower immune signature scores compared to *CD74*-hi group.

### Association of *CD74* and immune subtypes

The immune infiltration pattern within tumor microenvironment plays a pivotal role in immunotherapy efficiency. This concept, known as immune subtype, has been proved to be a predictive biomarker for ICI therapy in several studies [29–31]. Inspired by this, looking beyond *CD74*, we further identified three immune subtypes (Cluster 1, C1; Cluster 2, C2; Cluster 3, C3) based on the consensus clustering analysis of 22 immune cell- and immune-related signatures in our study (Fig. 3c). Each of the immune subtypes displayed distinct compositions of tumor-infiltrating immune cells and different expression levels of immune pathways. C1 (yellow light) presented moderate immune cell infiltration and immune pathways, while its median expression of dendritic cells, CT antigen, and senescence scores were highest in three clusters. In comparison, C2 (red light) had the relatively highest expression of immune cell infiltration and immune pathways (e.g., T cells, cytotoxic cells, and IFN-γ), defining as immune-inflamed subtypes. C3 (blue light) was nearly devoid of immune cells, exhibiting a “desert”-like subtype. The characteristics of these three immune phenotypes were similar to those reported in previous studies [32]. Accordingly, we inferred that C1, C2, and C3 tended to be immune-excluded subtypes, immune-inflamed subtypes, and immune-desert subtypes, respectively.

Next, we performed a correlation analysis between these distinct clusters and *CD74* expression status. *CD74* gene expression was significantly correlated with the immune subtypes (*p* = 0.0046) (Supplementary Fig. 4a). For baseline samples, *CD74*-hi tumors displayed C2 (7/10) and C1 subtypes (3/10), whereas *CD74*-lo tumors were distributed into C1 (5/11), C2 (1/11), and C3 (5/11) subtypes. No C3 subtype was found in *CD74*-hi tumors. After AK104 treatment, the transition from C1 or C3 to C2 was observed for three patients in *CD74*-lo group. The transition from C1 or C3 to C2 may explain the significant immune activation that occurred in *CD74*-lo group (Fig. 3b) and further support that AK104 therapy efficiently altered the tumor immune microenvironment and increased immune cell infiltration (Fig. 3d, Supplementary Fig. 4b). Notably, when we evaluated the *CD74* expression level of individual patients after AK104 treatment, we found the transition from *CD74*-lo to *CD74*-hi occurred in these three patients with subtype transition (Pts 9, 15, and 21; Supplementary Table 3). However, patients with C3 subtype who did not undergo subtype transition still exhibited low *CD74* gene expression after treatment (Pts 12–14; Supplementary Table 3). This result again demonstrated the association between CD74 expression and immune subtype.

Overall, our findings illustrate a distinct distribution of immune subtypes and responses to AK104 with different levels of *CD74* gene expression. *CD74*-hi tumors that presented with more immune-inflamed subtypes were able to respond effectively to AK104 immunotherapy.

### Visualization and validation of increased infiltrating T lymphocytes in *CD74*-hi tumors

**Fig. 4:**
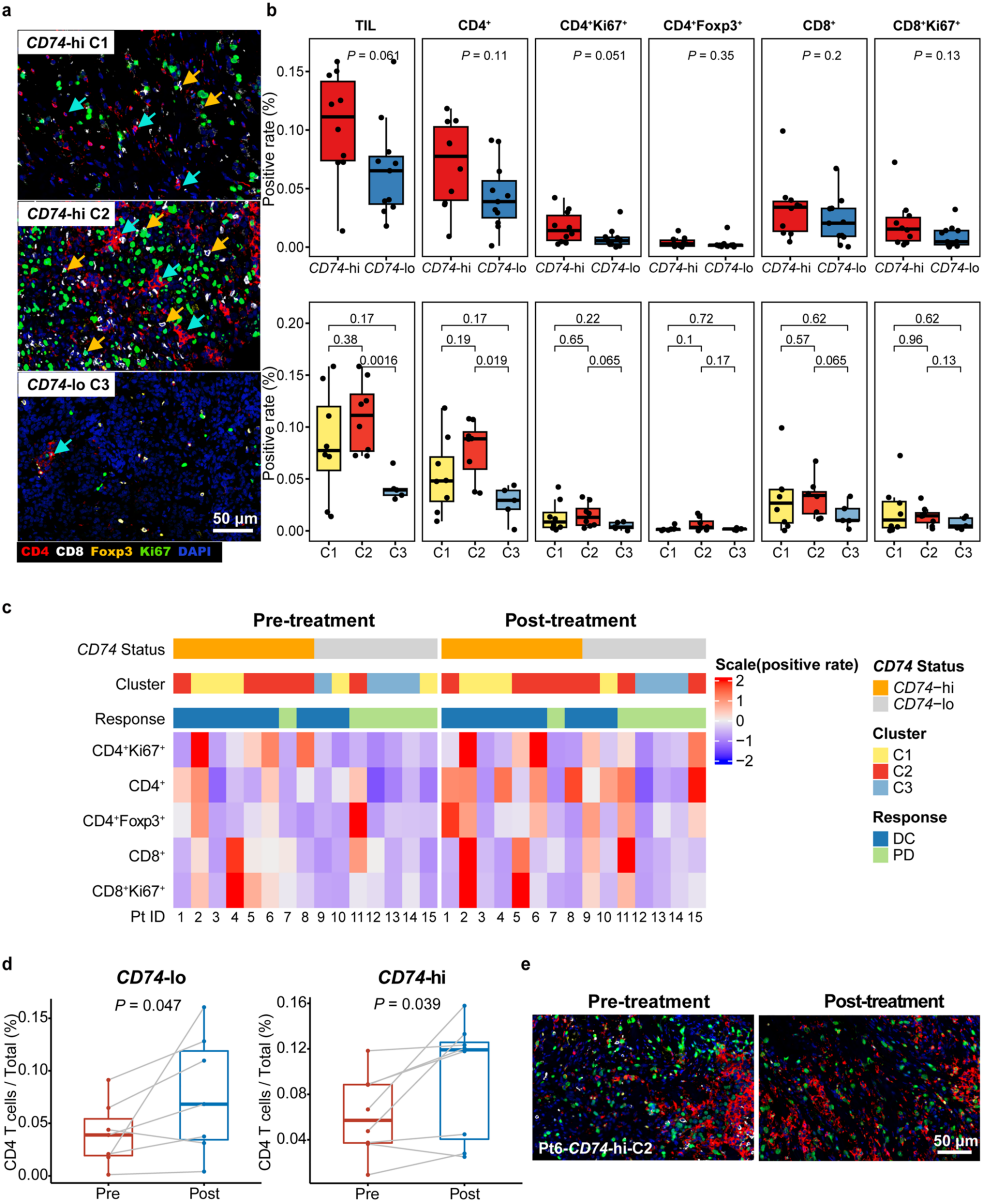
T cell infiltration profile analyzed by mIHC. **a** Representative mIHC images of baseline tissue sections in different patients. CD4 T cells (red), CD8 T cells (white), Treg marker (yellow) and Ki67 (green). Cyan arrows indicate CD4 T cells, and yellow arrows indicate CD8 T cells. **b** Proportion of different T cell subsets (% total) for baseline tumor specimens with *CD74*-hi and *CD74*-lo expression (top) or different immune subtypes (bottom). Tumor infiltrating lymphocytes (TILs, CD4^+^ & CD8^+^), CD4 T cells (CD4^+^), CD8 T cells (CD8^+^), proliferating CD4 T cells (CD4^+^Ki67^+^), proliferating CD8 T cells (CD8^+^Ki67^+^), and Tregs (CD4^+^Foxp3^+^). **c** Heatmap of different T cell subsets in pre- and post-treatment tumors. **d** Dynamic change of CD4 T cells before and after treatment in *CD74*-hi and *CD74*-lo groups. **e** Representative mIHC image of tissue sections at pre- and post-treatment time points for Pt6, who exhibited *CD74*-high status and C2 immune subtype. CD4 T cells (red), CD8 T cells (white), Treg marker (yellow), and Ki67 (green). *p*-values were calculated via Wilcoxon test. C1, cluster 1; C2, cluster 2; C3, cluster 3; *CD74*-hi, *CD74*-high; *CD74*-lo, *CD74*-low; DC, disease control; PD, progressive disease; Post, post-treatment; Pre, pre-treatment; Pt, patient

To gain further insight into the landscape of immune cells in tumor microenvironment, we employed mIHC staining on tissues collected before and after treatment to visualize the proportion and distribution of T lymphocytes. Consistent with the RNA results, more infiltrated T cells were observed in *CD74*-hi tumors than in *CD74*-lo tumors (Fig. 4a, b). The cumulative results showed that the ratios of CD4 T cells (*p* = 0.11), proliferating CD4 T cells (*p* = 0.051), and proliferating CD8 T cells (*p* = 0.13) were numerically higher in *CD74*-hi tumors (Fig. 4b, top). For immune subtypes, we also observed relatively higher T cells in C2 tumors, followed by C1 and C3 (Fig. 4b, bottom). After treatment, a significant increase of CD4 T cells was found in both *CD74*-hi and *CD74*-lo tumors as compared to CD8 T cells and Tregs (Fig. 4c, d). Representative images from three patients (Pts 1, 6, and 15) at both pre- and post-treatment time points showed a marked increase in CD4 T cell infiltration into the tumor following AK104 treatment (Fig. 4e, Supplementary Fig. 5). The significant increase in CD4 T cells post-treatment emphasized a critical role of CD4 T cell compartment in tumor control. Patients with C3 immune subtype (Pts 12–14) did not show any increase in T cell infiltrates, which verified the observations for the poor immune cell infiltrates of C3 tumors via NanoString. These results again suggest that AK104 efficiently alters the tumor immune microenvironment and promotes the infiltration of immune cells, which requires high levels of *CD74* gene expression.

### Predictive value of CD74 protein expression

**Fig. 5:**
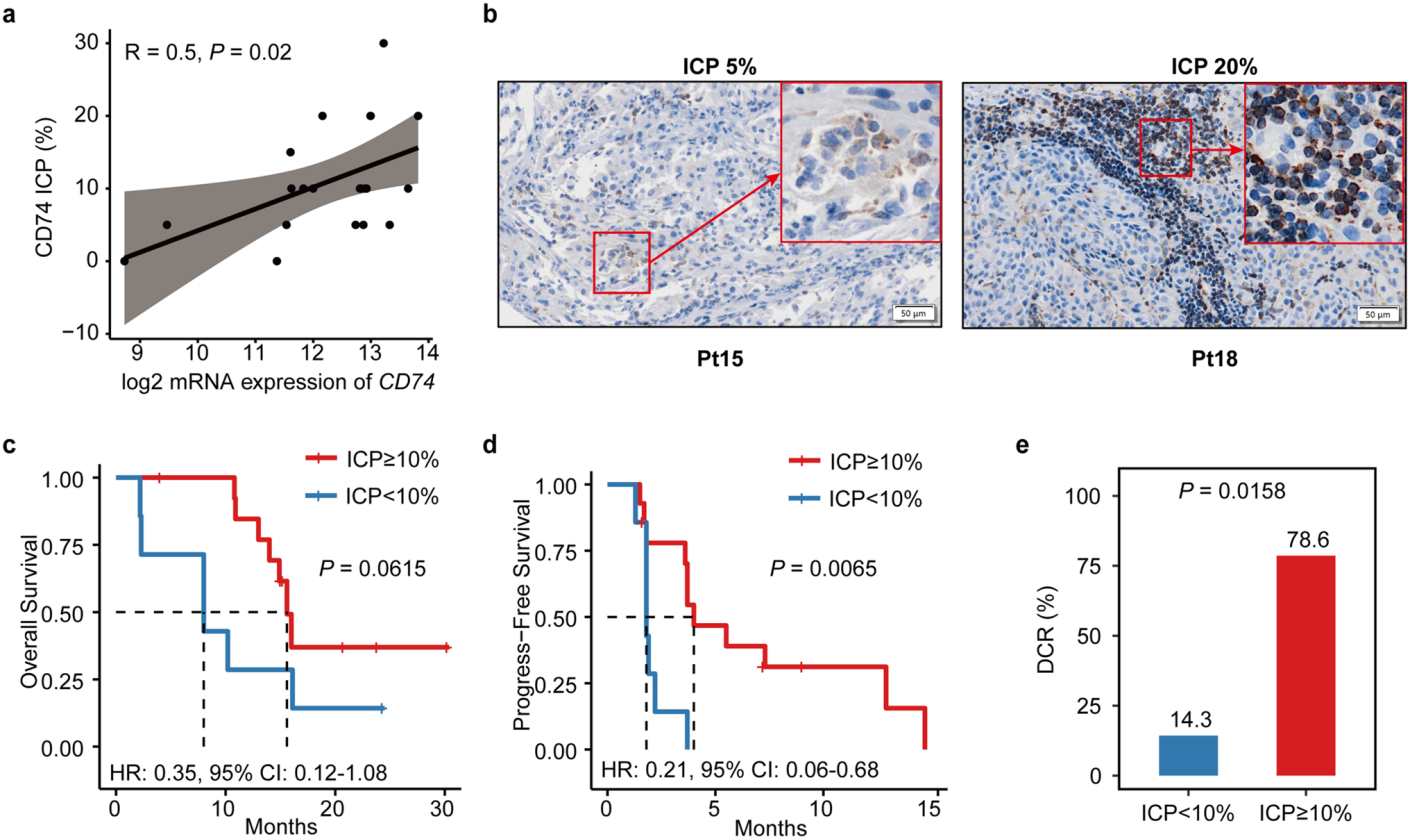
Predictive value of CD74 protein expression at 10% cut off value of ICP. **a** Correlation analysis between CD74 gene and protein expression. **b** Representative IHC images for CD74 (brown) in baseline tissue sections from two patients. Positive staining of cells is shown in enlarged inserts in the top right corner. OS (**c**) and PFS (**d**) curves of patients with high (ICP ≥ 10%, *n* = 14) and low (ICP < 10%, *n* = 7) protein expression of CD74. **e** Bar plot of DCR for CD74 ICP ≥ 10% (*n* = 14) and CD74 ICP < 10% (*n* = 7) groups. *p*-values were calculated via Pearson correlation coefficient test (**a**), Kaplan–Meier curves with the log-rank test (**c** and **d**), or Fisher’s exact test (**e**). DCR, disease control rate; ICP, the percentage of tumor area occupied by CD74 stained immune cells; Pt, patient

To further explore the predictive value of CD74, the protein expression of CD74 was visualized in tumor sections by IHC. Correlation analysis showed a positive relationship between protein and gene expression of CD74 (Pearson correlation: *R* = 0.5, *p* = 0.02) (Fig. 5a). Representative examples of different CD74 protein expressions are shown in Fig. 5b. CD74 is predominantly expressed in the cytoplasm and membrane of immune cells and tumor cells. PFS and OS survival benefits were observed in patients with high CD74 protein expression (ICP ≥ 10%, *n* = 14) compared to those with low CD74 protein expression (ICP < 10%, *n* = 7), showing significance for PFS (HR = 0.21, 95% CI 0.06–0.68, *p* = 0.0065; Fig. 5c, d). Comparison of DCR also revealed a significantly higher DCR in CD74 ICP ≥ 10% group (*p* = 0.0158; Fig. 5e).

## Discussion

This study is the first investigation to evaluate the predictive biomarker in patients with advanced solid tumors treated with cadonilimab (AK104). Our work illustrated that the gene and protein expressions of CD74 were associated with clinical outcomes, immune subtypes in tumor microenvironment, and immune responses to ICIs treatment. Specifically, *CD74* expression was positively associated with immune infiltration in advanced solid tumor microenvironment, consistent with previous studies in patients with glioma, hepatocellular carcinoma, melanoma, and malignant pleural mesothelioma [33–36]. Tumors with high expression level of *CD74* tend to display an immune-inflamed subtype, which shows the potential in anti-tumor immune responses [37]. Moreover, this is also the initial report to explore the modulation of tumor microenvironment by AK104 in clinic therapy. In previous preclinical research, we reported that AK104 efficiently enhanced the release of IL-2 and IFN-γ in vitro [38], which will promote T cell activation. Similarly, we did observe an increase of immune cells and enriched IFN-γ pathway in this clinical trial. Specifically, a greatly increase of immune cells was found in *CD74*-lo group compared to *CD74*-hi group. This could be explained by the much lower level of immune cells of *CD74*-lo group than that in *CD74*-hi at baseline, and the inflamed subtype transition occurred in *CD74*-lo group. Taken together, our results demonstrated that AK104 could efficiently improve tumor immune microenvironment, and CD74 could serve as a predictive biomarker.

Antigen presentation is an essential pathway for an efficient treatment response to ICI therapy, and CD74 has been implicated to this process in immune-oncology. Previous studies revealed that CD74 participates in HLA-II antigen presentation and plays a physiological function in antigen cross-presentation, inducing the priming of CD4 T cell and CD8 T cell immunity [26, 27]. The disruption of HLA-I antigen processing and presentation machinery mediates immune evasion and serves as a mechanism of acquired resistance to ICIs in tumors [39]. The correlation of CD74 and HLA II molecules as well as immune cell infiltration has been widely reported [40, 41]. In this study, we noticed that tumors with high gene expression levels of *CD74* were characterized by the enrichment of HLA-II/I, B cell function, T cell function, and NK cell function gene sets compared to those tumors with low *CD74* gene expression. We speculated that high *CD74* expression was attributed to high tumoral infiltration of immune cells, resulting in further efficient HLA antigen presentation and anti-tumor response. This hypothesis was further verified as we observed increased *CD74* expression in patients with inflamed subtype transition.

Beyond antigen presentation, cell surface CD74 also acts as a receptor for migration inhibitory factor (MIF). It has been reported that CD74-MIF signaling pathway regulates PD-L1 expression and promotes tumor cells escape from immune surveillance [42]. Notably, defined PD-L1 cutoffs based on different interpretations (e.g., combined positive score [CPS] ≥ 1, tumor proportion score [TPS] ≥ 1%) have been approved by the US Food and Drug Administration to guide the selection of patients to receive anti-PD-1 (e.g., nivolumab and pembrolizumab) or anti-PD-L1 antibodies [43–45]. Patients with high PD-L1 expression in tumor tissue seem more likely to benefit from anti-PD-1/PD-L1 antibody therapy. Our work revealed that *CD274* expression was positively correlated with *CD74* expression, as was *CTLA4* expression. Similar results were observed when we analyzed the data from TCGA. Patients with high *CD74* gene expression and protein expression (ICP ≥ 10%) effective clinical response and prolonged survival following AK104 treatment, suggesting that CD74 has potential predictive performance in AK104 cancer therapy. Previous data have shown that PD-L1 protein expression is significantly correlated with CD74 protein expression [40]. Despite the absence of PD-L1 protein expression data due to limited tumor tissue materials in this study, we inferred that the predictive value of CD74 protein expression might also be contributed by PD-L1 expression.

To date, there is no uniform answer to the role of CD74 in tumors, as it exhibits dramatic biological differences owing to its different location patterns and derived cell types. At steady state, a small fraction of CD74 is expressed on the cell surface independently of MHC-II processing pathway [46]. Cytoplasmic CD74 expressed on APCs functions as an antigen-presenting partner, whereas the overexpression of cell surface CD74 on tumor cells or immune cells serves as MIF receptor, promoting the release of pro-inflammatory and pro-angiogenic factors along with CD44 and CXCR4 ligands [47]. CD74 expression on tumor cells or total cells has been proved as a favorable prognostic biomarker. In MESO and breast cancer, high CD74 tumor cell expression at different cutoffs of histochemistry score (H-score) (MESO: H-score ≥ 400; breast cancer: H-score ≥ 20) was positively associated with longer survival [34, 36]. In other studies, high proportion of CD74 positive cells predicted favorable prognosis in patients [35]. Instead, we observed a high CD74 expression score using immune cells (ICP ≥ 10%) correlated with clinical benefits in our current study. Moreover, CD74 expression scored by TPS, IPS, or CPS failed to associate with clinical response based on ROC curve analysis in this study (Supplementary Fig. 6). This point needs to be investigated further. The difference we observed raised an interesting question about the previously reported performance of CD74 protein expression on tumor cells and/or total cells. Further study is warranted to identify an optimal stratification in outcome prediction based on tumor cells and/or immune cells, and specific cancer types should be considered.

The limitations of this study include its small sample size, single arm design, and multiple cancer types. Additional examinations would help provide a better understanding of the underlying mechanism of CD74 in ICI therapy. The research on the correlation between CD74 protein expression and clinical outcomes in specific cancer types (e.g., NSCLC, cervical cancer) is ongoing.

In summary, our analyses highlighted the potential predictive value of CD74 in patients with advanced solid tumors after AK104 treatment. This hypothesis needs to be validated in another prospective study using the predefined cutoffs for CD74 assessment to better understand its predictive value in the context of immuno-oncology therapy.

### Supplementary Information

Below is the link to the electronic supplementary material.

Supplementary file1 (DOCX 6386 KB)

## Data Availability

Data in this study are available within the article and its supplementary data files. The raw data is available upon reasonable request from the corresponding author.

## Abbreviations

C1: Cluster 1
C2: Cluster 2
C3: Cluster 3
DC: Disease control
DEG: Differential expression gene
ICP: Immune cell present, the percentage of tumor area occupied by CD74 staining immune cells
mIHC: Multiplex immunohistochemistry
