# Supplementary material for: CD74 is associated with inflamed tumor immune microenvironment and predicts responsiveness to PD-1/CTLA-4 bispecific antibody in patients with solid tumors
Source: Cancer Immunol Immunother. 2024 Jan 27;73(2):36. doi: 10.1007/s00262-023-03604-2 (PMC10822011; doi:10.1007/s00262-023-03604-2)
Supplement: Supplementary file 1 — Supplementary file1 (DOCX 6386 KB) [file 262_2023_3604_MOESM1_ESM.docx]

**Supplementary materials**

Cancer Immunology, Immunotherapy (submitted in 2023) Jianghua Wang et al.

**Supplementary Tables**

**Supplementary Table 1**

| **Gene** | **Group** | **PFS** | | | |
| --- | --- | --- | --- | --- | --- |
|  |  | **median(months)** | **HR** | **95% CI** | **P value** |
| CD74 | Low (n=24) | 3.0 | 0.36 | 0.18-0.74 | 0.0040 |
|  | High (n=24) | 25.1 |  |  |  |
| CREB5 | Low (n=24) | 5.8 | 1.55 | 0.81-2.97 | 0.1860 |
|  | High (n=24) | 2.8 |  |  |  |
| CD200 | Low (n=24) | 4.9 | 1.13 | 0.59-2.16 | 0.7123 |
|  | High (n=24) | 3.3 |  |  |  |
| ST6GAL1 | Low (n=24) | 7.6 | 1.96 | 1.01-3.79 | 0.0420 |
|  | High (n=24) | 3.1 |  |  |  |
| **Gene** | **Group** | **OS** | | | |
|  |  | **median(months)** | **HR** | **95% CI** | **P value** |
| CD74 | Low (n=24) | 10.1 | 0.49 | 0.24-1.01 | 0.0494 |
|  | High (n=24) | 31.3 |  |  |  |
| CREB5 | Low (n=24) | 28.2 | 2.01 | 0.99-4.09 | 0.0501 |
|  | High (n=24) | 8.1 |  |  |  |
| CD200 | Low (n=24) | 22.9 | 1.46 | 0.72-2.97 | 0.2930 |
|  | High (n=24) | 14.4 |  |  |  |
| ST6GAL1 | Low (n=24) | 32.3 | 2.40 | 1.15-5.03 | 0.0166 |
|  | High (n=24) | 10.1 |  |  |  |

Survival analysis based on four gene expressions in Liu's cohort

**Supplementary Table 2**

| **Gene** | **Group** | **PFS** | | | |
| --- | --- | --- | --- | --- | --- |
|  |  | **median(months)** | **HR** | **95% CI** | **P value** |
| CD74 | Low (n=23) | 2.8 | 0.51 | 0.25-1.03 | 0.0578 |
|  | High (n=24) | 6.5 |  |  |  |
| CREB5 | Low (n=23) | 3.2 | 0.87 | 0.44-1.71 | 0.6940 |
|  | High (n=24) | 3.2 |  |  |  |
| CD200 | Low (n=23) | 3.2 | 0.91 | 0.46-1.81 | 0.7932 |
|  | High (n=24) | 3.5 |  |  |  |
| ST6GAL1 | Low (n=23) | 3.2 | 0.72 | 0.36-1.44 | 0.3496 |
|  | High (n=24) | 3.8 |  |  |  |

Survival analysis based on four gene expressions in Prat's cohort

**Supplementary Table 3**

| **Patients** | **Cancer Type** | ***CD74* Status** | | **Cluster** | |
| --- | --- | --- | --- | --- | --- |
|  |  | **Pre** | **Post** | **Pre** | **Post** |
| Pt1 | MESO | *CD74*-hi | *CD74*-hi | C2 | C2 |
| Pt2 | MESO | *CD74*-hi | *CD74*-hi | C1 | C1 |
| Pt3 | HNSCC | *CD74*-hi | *CD74*-lo | C1 | C1 |
| Pt4 | MESO | *CD74*-hi | *CD74*-hi | C1 | C1 |
| Pt5 | TNBC | *CD74*-hi | *CD74*-hi | C2 | C2 |
| Pt6 | MESO | *CD74*-hi | *CD74*-hi | C2 | C2 |
| Pt7 | NSCLC | *CD74*-hi | *CD74*-hi | C2 | C2 |
| Pt8 | MESO | *CD74*-hi | *CD74*-hi | C2 | C2 |
| Pt9 | Sarcoma | *CD74*-lo | *CD74*-hi | C3 | C2 |
| Pt10 | HCC | *CD74*-lo | *CD74*-lo | C1 | C1 |
| Pt11 | GC | *CD74*-lo | *CD74*-hi | C2 | C2 |
| Pt12 | SCLC | *CD74*-lo | *CD74*-lo | C3 | C3 |
| Pt13 | Sarcoma | *CD74*-lo | *CD74*-lo | C3 | C3 |
| Pt14 | OC | *CD74*-lo | *CD74*-lo | C3 | C3 |
| Pt15 | RCC | *CD74*-lo | *CD74*-hi | C1 | C2 |
| Pt16 | NSCLC | *CD74*-lo |  | C3 |  |
| Pt17 | GEJ cancer | *CD74*-hi | *CD74*-lo | C2 | C2 |
| Pt18 | MESO | *CD74*-hi |  | C2 |  |
| Pt19 | Sarcoma | *CD74*-lo |  | C1 |  |
| Pt20 | TNBC | *CD74*-lo |  | C1 |  |
| Pt21 | CRC | *CD74*-lo | *CD74*-hi | C1 | C2 |
| Abbreviations: C1, cluster 1; C2, cluster 2; C3, cluster 3; *CD74*-hi, *CD74*-high; *CD74*-lo, *CD74*-low; CRC, colorectal cancer; GC, gastric cancer; GEJ cancer, gastroesophageal junction (GEJ) cancer; HCC, hepatocellular cancer; HNSCC, head and neck squamous cell cancer; MESO, mesothelioma; NSCLC, non-small cell lung cancer; OC, ovarian cancer; Post, post-treatment; Pre, pre-treatment; Pt, patient; RCC, renal cell cancer; SCLC, small-cell lung cancer; TNBC, triple negative breast cancer | | | | | |

Cluster information of each patients before and after AK104 treatment

**Supplementary Figures**

**Supplementary Figure 1**

Schematic illustration of biomarker analysis in Cadonilimab (AK104) clinical trial (NCT03261011). Patients enrolled in the pharmacodynamic confirmation cohort underwent tumor biopsy at screening and day 29 post-treatment (pre-dose). Tissues were subsequently analyzed by NanoString nCounter and multiplex immunohistochemistry to explore gene expression profiles (GEP) and tumor-infiltrating lymphocytes, respectively, aiming at identifying potential predictive biomarkers and studying the immune mechanism for AK104 immunotherapy. FFPE, formalin-fixed paraffin-embedded; i.v., intravenous injection

**Supplementary Figure 2**


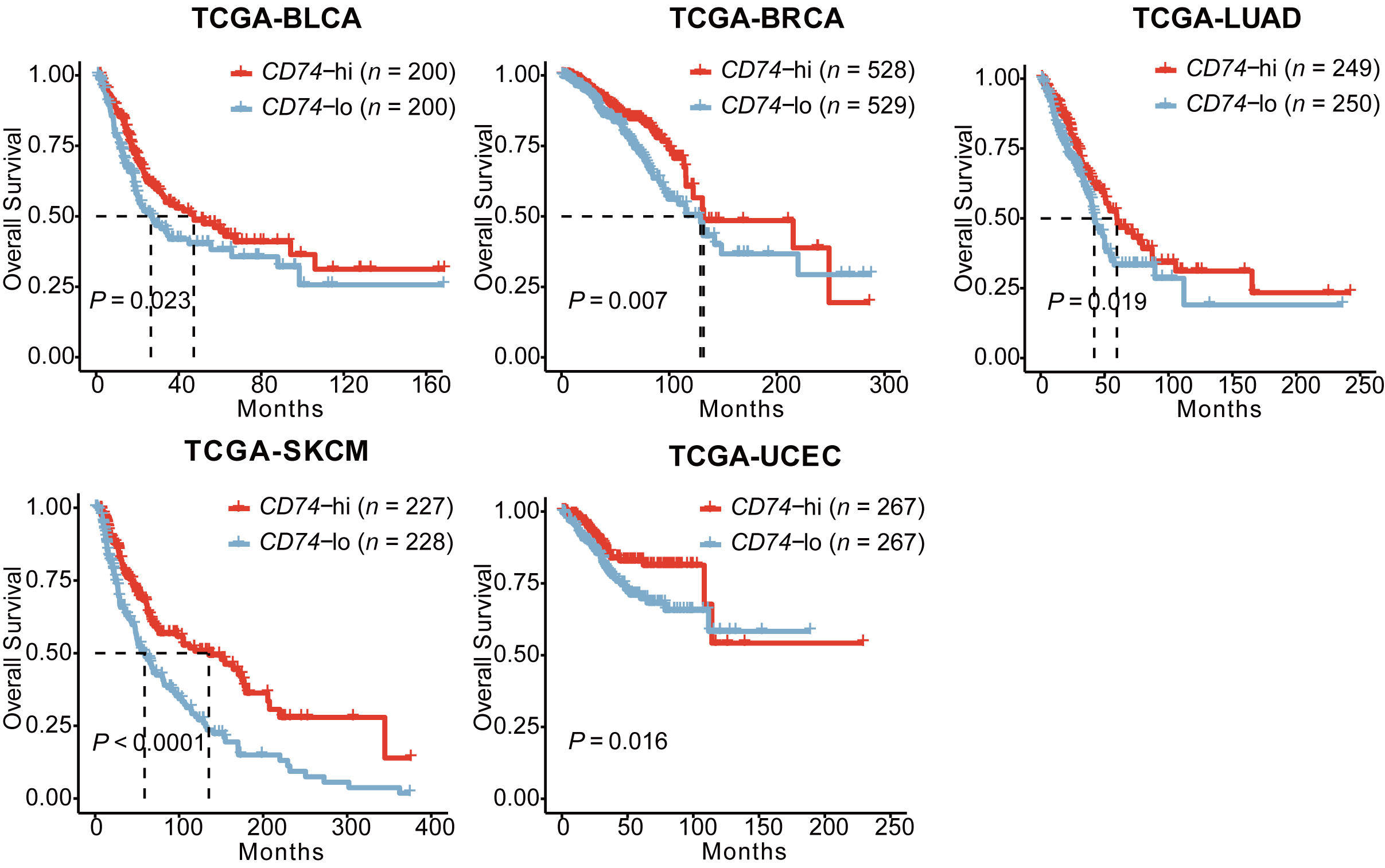


Correlation between *CD74* gene expression and overall survival in TCGA cohort. P-values were calculated via Wald test. BLCA: bladder urothelial carcinoma; BRCA: breast invasive carcinoma; *CD74*-hi, *CD74*-high; *CD74*-lo, *CD74*-low; ECOG, Eastern Cooperative Oncology Group; F, female; LUAD: lung adenocarcinoma; M, male; SKCM: skin cutaneous melanoma; TCGA, The Cancer Genome Atlas; UCEC: uterine corpus endometrial carcinoma

**Supplementary Figure 3**


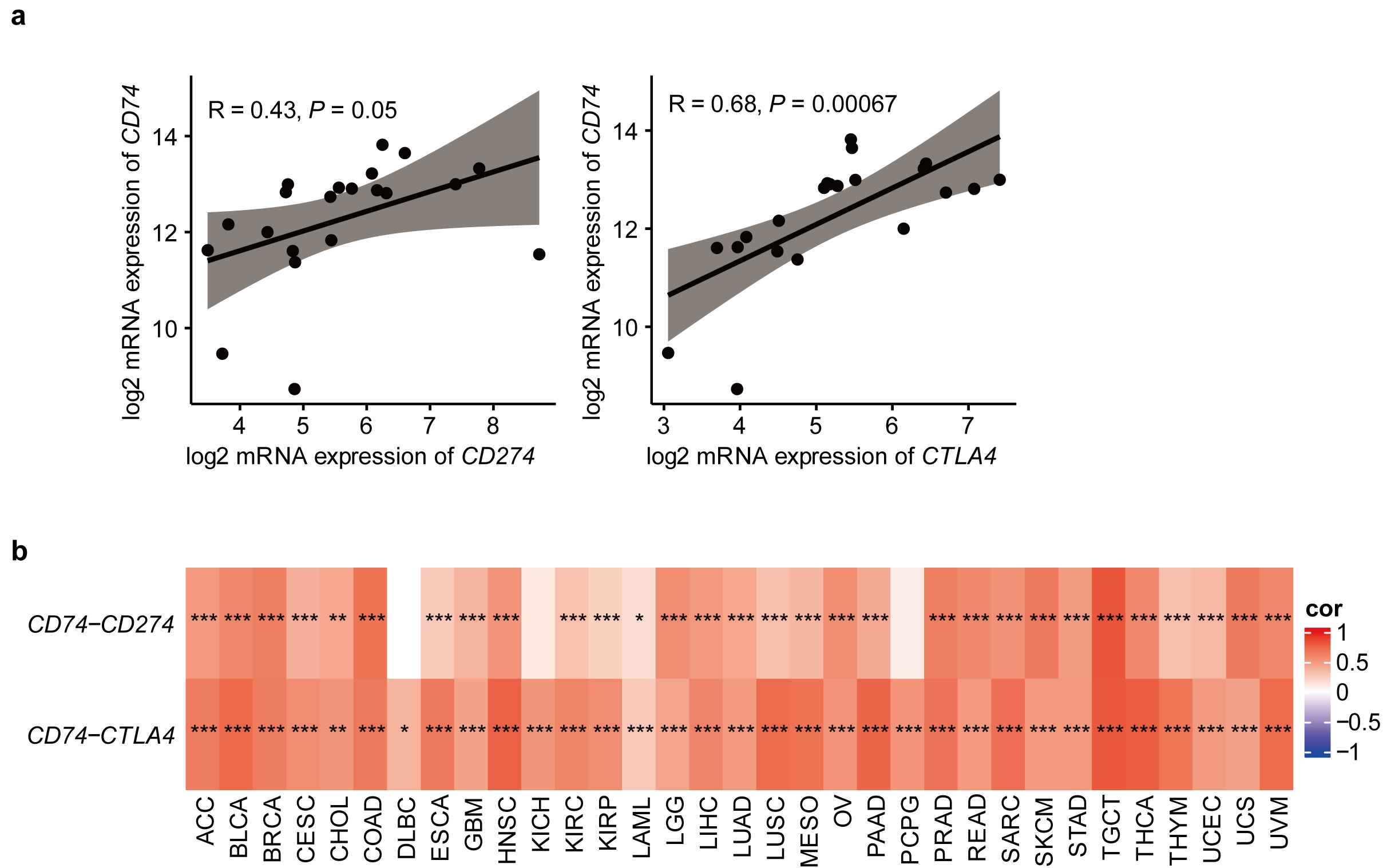


Correlation of *CD74* and *CD274* or *CTLA4* in AK104-treated cohort (**a**) and TCGA cohort (**b**). P-values were calculated via Pearson correlation coefficient test. *p<0.05, **p<0.01, ***p<0.001. ACC, adrenocortical carcinoma; BLCA, bladder urothelial carcinoma; BRCA, breast invasive carcinoma; CESC, cervical squamous cell carcinoma and endocervical adenocarcinoma; CHOL, cholangiocarcinoma; COAD, colon adenocarcinoma; Cor, Correlation index; DLBC, diffuse large B-cell lymphoma; ESCA, esophageal carcinoma; GBM, glioblastoma carcinoma; HNSC, head and neck squamous cell carcinoma; KICH, kidney chromophobe; KIRC, kidney renal clear cell carcinoma; KIRP, kidney renal papillary cell carcinoma; LAML, acute myeloid leukemia; LGG, brain lower grade glioma; LIHC, liver hepatocellular carcinoma; LUAD, lung adenocarcinoma; LUSC, lung squamous cell carcinoma; MESO, mesothelioma; OV, ovarian serous cystadenocarcinoma; PAAD, pancreatic adenocarcinoma; PCPG, pheochromocytoma and paraganglioma; PRAD, prostate adenocarcinoma; READ, rectum adenocarcinoma; SARC, sarcoma; SKCM, skin cutaneous melanoma; STAD, stomach adenocarcinoma; TGCT, testicular germ cell tumors; THCA, thyroid carcinoma; THYM, thymoma; UCEC, uterine corpus endometrial carcinoma; UCS, uterine carcinosarcoma; UVM, uveal melanoma

**Supplementary Figure 4**


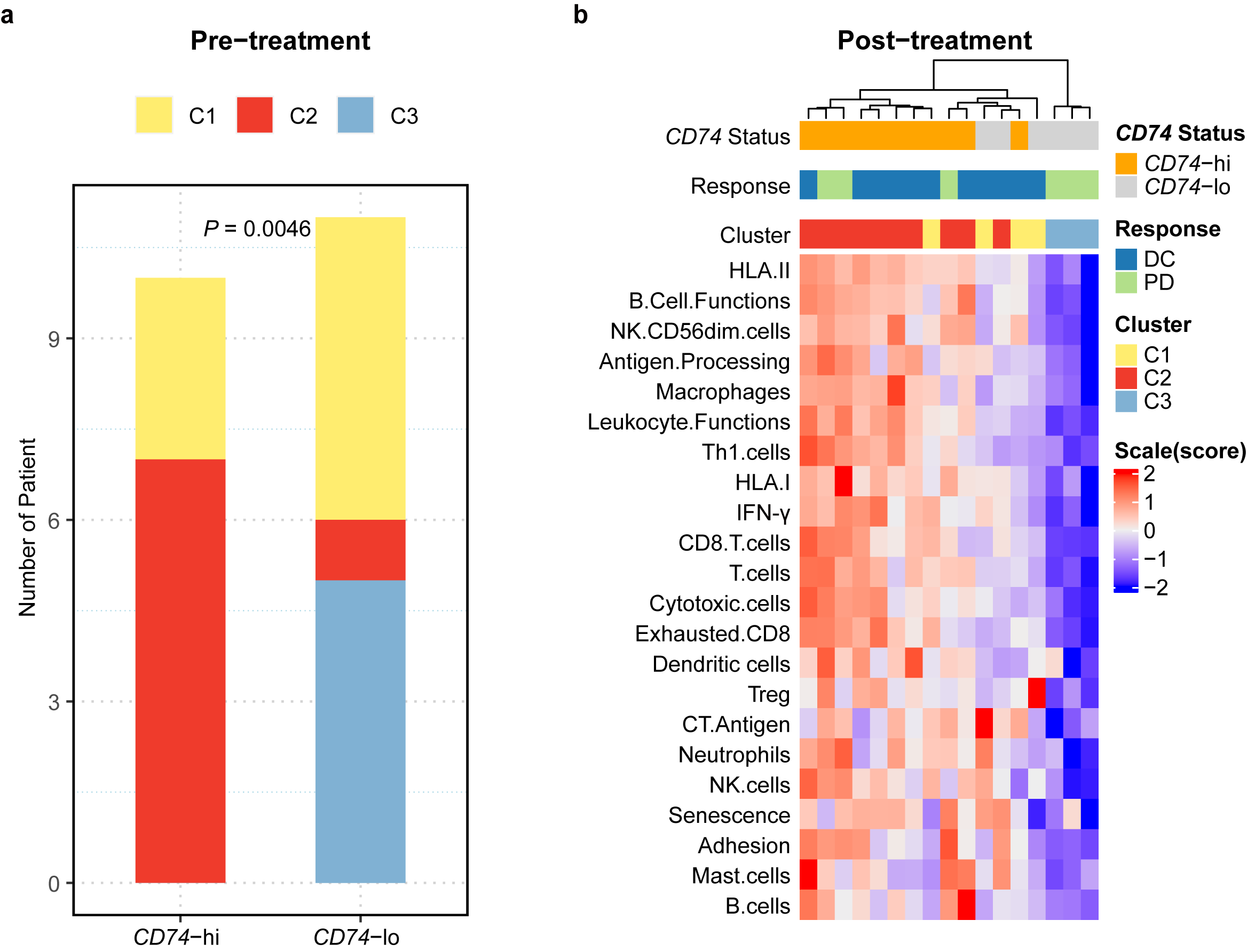


Cluster information for pre- and post-treatment tumors. (**a**) Bar plot of distribution of immune subtypes in *CD74* gene expression status (*CD74*-hi, *n*=10; *CD74*-lo, *n*=11) at pre-treatment time point. (**b**) Immune subtype clustering analysis for AK104-treated tumor samples based on 22 immune-related signatures. P-values were calculated via Fisher’s exact test. C1, cluster 1; C2, cluster 2; C3, cluster 3; *CD74*-hi, *CD74*-high; *CD74*-lo, *CD74*-low; CT, cancer/testis; DC, disease control; HLA, human leukocyte antigen; IFN-γ, interferon-γ; NK, natural killer; PD, progressive disease; Th1 cells, type 1 T helper cells; Treg, regulatory T cell

**Supplementary Figure 5**


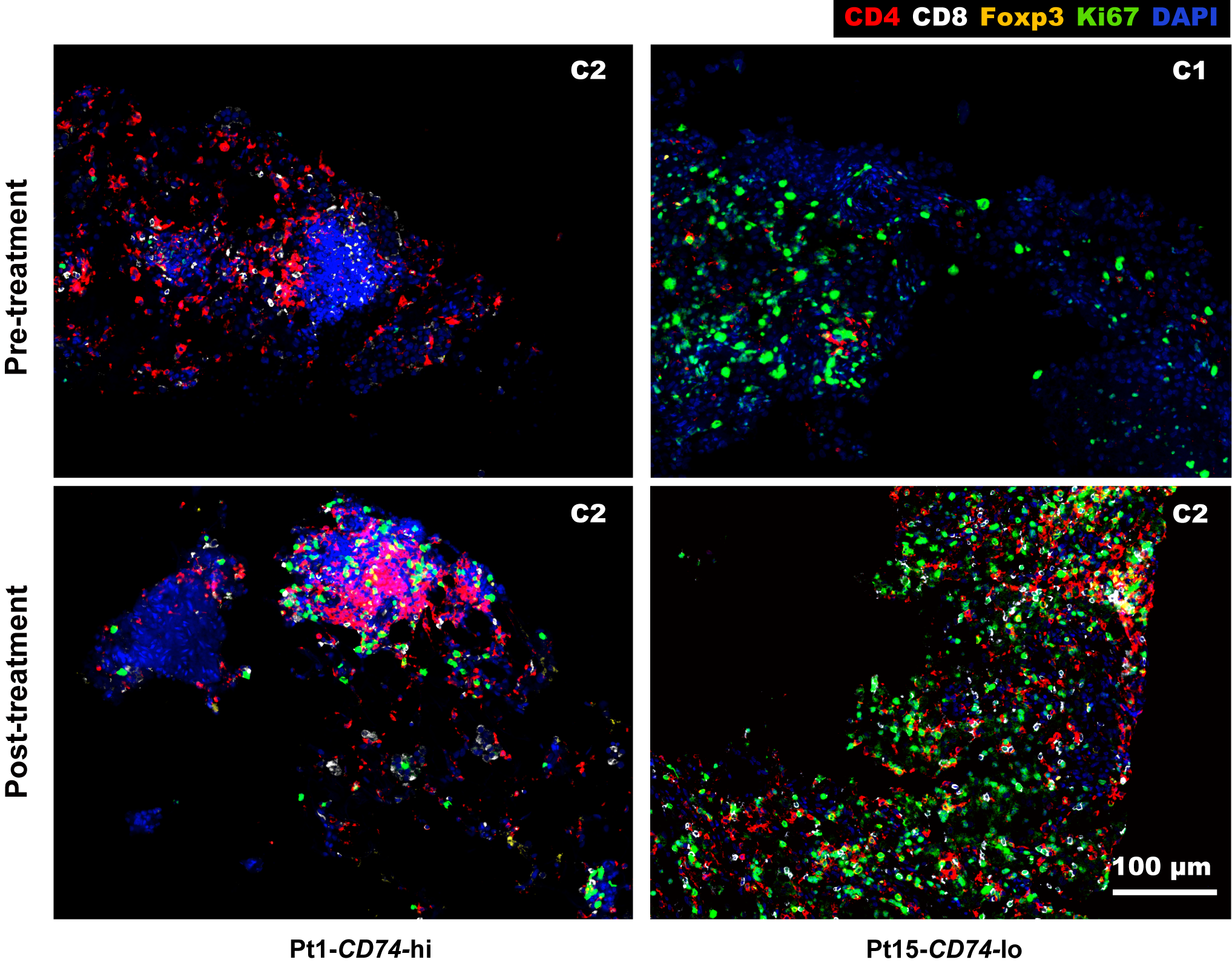


Representative mIHC images of pre- and post-treatment tissue sections for Pt1 (*CD74*-hi, C2) and Pt15 (*CD74*-lo, C1). CD4 T cells (red), CD8 T cells (white), Treg marker (yellow), and Ki67 (green). C1, cluster 1; C2, cluster 2; *CD74*-hi, *CD74*-high; *CD74*-lo, *CD74*-low

**Supplementary Figure 6**


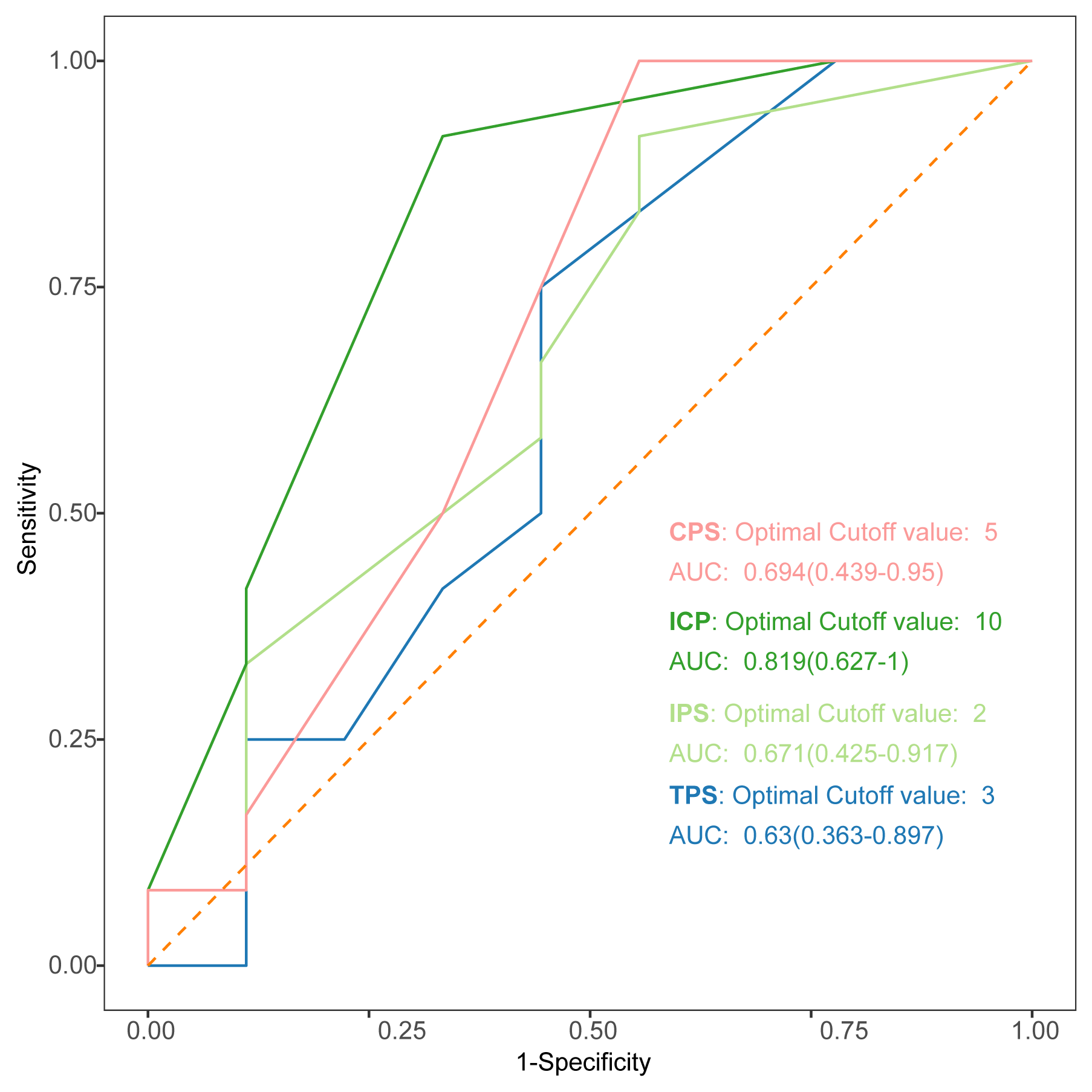


ROC curve of CD74 expression measured by different scoring systems. Combined positive score (CPS), the number of CD74 stained cells (tumor and immune cells) divided by the total number of viable tumor cells, multiplied by 100. Immune cell present (ICP), the percentage of tumor area occupied by CD74 stained immune cells. Immune cell proportion score (IPS), the percentage of CD74 stained tumor-associated immune cells in total tumor-infiltrating immune cells. Tumor cell proportion score (TPS), the percentage of CD74 stained tumor cells in total viable tumor cells
